# The long-term effectiveness of the International Child Development Programme (ICDP) implemented as a community-wide parenting programme

**DOI:** 10.1080/17405629.2014.950219

**Published:** 2014-08-21

**Authors:** Ane-Marthe Solheim Skar, Stephen von Tetzchner, Claudine Clucas, Lorraine Sherr

**Affiliations:** ^a^ Department of Psychology, University of Oslo, Oslo, Norway; ^b^ Department of Infection & Population Health, University College London, London, UK

**Keywords:** Early child development, Caregiver guidance, Community intervention, Long term follow-up, ICDP

## Abstract

Short-term effectiveness of the International Child Development Programme (ICDP) for parents in the general population has been studied. The aim of this paper was to investigate the longer term impact of the ICDP programme on parents looking for sustained changes 6–12 months after the programme. For this, a non-clinical caregiver group attending the ICDP programme (*N* = 79) and a non-attending comparison group (*N* = 62) completed questionnaires on parenting, psychosocial functioning, and child difficulties before, on completion and 6–12 months after the ICDP programme. Analyses compare changes in scores over time. The results revealed that the ICDP group showed significantly improved scores on parenting measures, less loneliness, and trends towards improved self-efficacy compared to the comparison group 6–12 months after programme completion. The ICDP group also reported that their children spent significantly less time on television and computer games and a trend towards fewer child difficulties. Key positive effects sustained over time but at a somewhat lower level, supporting community-wide implementation of ICDP as a general parenting programme. It is concluded that more intensive training with follow-up sessions should be considered to sustain and boost initial gains.

Parenting strategies and relationships are assumed to influence all aspects of child development (O'Connor & Scott, 2007). Research on programmes aimed at strengthening familial relationships and supporting child development has demonstrated that changes in parents' child management strategies may contribute to positive child development (e.g. Eddy & Chamberlain, 2000; Sandler, Schoenfelder, Wolchik, & MacKinnon, 2011). Parent training has proved to be effective in diverse contexts with a variety of child groups, including children in low-income communities (Gross et al., 2003), children with conduct and attention problems (Barlow & Stewart-Brown, 2000), and non-clinical populations (Sherr, Skar, Clucas, von Tetzchner, & Hundeide, 2014).

Long-term impact has been demonstrated in longitudinal studies (Sandler et al., 2011). In one study of 207 parents attending a 12-week parental course, significant increased self-efficacy and less coercive discipline one year after the intervention was found (Gross et al., 2003). For 238 newly divorced mothers and their sons (mean age 7.8 years) attending 14 group sessions of Parent Management Training, a cycle of change was noted whereby the parental intervention was associated with (1) more effective/positive parenting practices, and (2) reduced child conduct difficulties and (3) reduced parental depression (DeGarmo, Patterson, & Forgatch, 2004). Another study found higher effect sizes in the months following an intervention compared to immediately after, supporting the notion that change processes might need time to manifest (Vitaro, Brendgen, & Tremblay, 2001). In a review of 48 group implemented parenting programmes, significant short-term improvements were found for depression, anxiety, stress, anger, guilt, confidence and partner satisfaction; however, only stress and confidence continued to be significant 6 months later, and none were significant 1 year after (Barlow, Smailagic, Huband, Roloff, & Bennett, 2012). Most of these studies included selected groups of parents and behaviour-oriented interventions focusing on identified child difficulties. Less is known about the long-term effects of general population programmes for non-clinical parent populations (Hiscock et al., 2008). Evaluations of community-wide implementation of the evidence-based Triple P – Positive Parenting Program, used in about 20 countries, suggests reductions in disruptive child behaviour, dysfunctional parenting and co-parenting conflicts, and improved parental mental health 6–12 months after programme implementation (Dean, Myors, & Evans, 2003), and reductions in dysfunctional parenting and internalizing and externalizing child behaviours two years after (Hahlweg, Heinrichs, Kurschel, Bertran, & Naumann, 2010).

The International Child Development Programme (ICDP) is an interactive psychosocial programme directed towards parents and other caregivers, used in about 30 countries in cooperation with a variety of governmental and independent organizations. Several student theses and internal reports support the ICDP as effective in promoting positive parenting practices, increasing adjustment and strengthening familial relationships (ICDP, 2014), yet the programme is not yet rated as evidence-based due to a lack of research on the long-term effects and effects across contexts and receivers (Ungsinn, 2014). The short-term effects of attending universal ICDP groups have been investigated in Norway (Sherr et al., 2014). The ICDP group showed more positive attitudes towards child management and improved parenting strategies, and reported lower impact of child difficulties after attending ICDP groups. Parents with low initial scores benefited most. The impact of the ICDP programme has also been investigated in a community-sample in Mozambique (Skar, Sherr, Clucas, & von Tetzchner, 2014). The ICDP group reported more commitment and caring for the child, less severe physical punishment, less parental mental health difficulties, higher life quality and fewer child conduct problems than the comparison group. Time since programme attendance (0.5–5 years) did not seem to influence the outcomes, suggesting that the influence of the programme was sustained over time. However, this study included post-intervention comparisons only.

The current study investigates the impact of attending the ICDP programme 6–12 months after the group meetings to see if the short-term benefits are sustained or new benefits emerge. It was hypothesized that the changes in parenting strategies and in the parents' reported strengths and difficulties in the child after attending the ICDP programme would be maintained.

## METHOD

The study used a two-group design with one group attending the ICDP programme (*N* = 79) and a non-attending comparison group (*N* = 62) completing questionnaires before and immediately after the ICDP, and then again 6–12 months after the last group meeting.

### The ICDP programme: Content and implementation

The ICDP was developed in the 1980s by Profs Hundeide and Rye at the University of Oslo, with international colleagues, and registered as a foundation in Norway in 1992 (Hundeide & Rye, 2010). ICDP builds upon the Convention on the Rights of the Child (United Nations Human Rights, 2014) and humanistic psychology. The programme is formulated as three dialogues containing eight guidelines for good interaction: the emotional dialogue (showing loving feelings, following the child's lead, having good personal dialogue with the child, praising and acknowledging the child), the comprehension dialogue (helping the child to focus attention, giving meaning and enthusiasm for the child's experiences, expanding and enriching the child's experiences), and the regulative dialogue (regulating the child's actions step-by-step). The dialogues are influenced by research on attachment, pedagogical interaction and regulation (Hundeide, 2001, 2010a).

The ICDP approach is facilitative and skill based rather than instructive, and is thought to be culturally flexible by being grounded in the cultural experiences of the caregivers. The programme is delivered in a group format where the attendees share, discuss and reflect on the emotional, comprehension and regulative components of child rearing, followed by home assignments to try out new learning, and subsequently share their experiences with the group (Hundeide, 2010b). In Norway, the ICDP programme is available to all parents on a voluntary basis and implemented by the Ministry of Children, Equality and Social Inclusion. Staff are trained as ICDP facilitators and lead the group meetings with the support of an ICDP manual that provides theoretical background of ICDP (Hundeide, 2010a), and practical operational advices (Hundeide, 2010b). The groups usually consist of 5–10 caregivers attending eight weekly two-hour sessions (Sherr et al., 2014).

### Participants

Project participants were recruited between October 2008 and March 2010 among parents attending newly initiated ICDP groups based on national availability in Norway (see Sherr et al., 2014). At the first ICDP meeting, attendees who wanted to participate gave consent. A comparison group not attending ICDP or similar programmes was recruited from kindergartens and child health centres in socio-economical matched areas to control for the passage of time and parenting experiential learning. Follow-up questionnaires were sent by mail 6–12 months later with one reminder. Of the 141 ICDP and 79 comparison parents who completed questionnaires before and after the ICDP programme, 79 (56.03%) in the ICDP group and 62 (78.48%) in the comparison group returned follow-up questionnaires. Only data from participants with full follow-up are used in the present analysis.

At the first questionnaire completion (baseline), the mean age of ICDP caregivers was 34.2 years (SD = 6.87, range 23–60), with average 2.0 children (SD = 1.00, range 1–6), having a mean of 3.5 people in the home (SD = 1.24, range 1–6). The focus child (closest in age to 4) was 3.6 years old (SD = 2.29, range 50–12.0). The mean age of the comparison group was 34.8 years (SD = 5.50, range 24–47), with an average of 1.8 children (SD = .73, range 1–4), and there were an average of 3.4 people in the home (SD = 1.20, range 1–6). The focus child was 3.4 years old (SD = 1.85, range 25–11.0). The groups did not differ significantly on any of these variables or any other demographic variable, except education. Caregivers in the comparison group were significantly more likely to have higher education, and this was adjusted for in the subsequent analysis (see Table 1).

**Table 1:** Characteristics of caregivers in the ICDP group and the comparison group (*N* = 141)

	ICDP (*N* = 79)	Comparison (*N* = 62)	
Variable	*N*	*N*	*p*
Gender			.840
Female	62	47	
Male	17	14	
Civil status			.136^a^
Married/partner	75	58	
Separated/divorced	1	2	
Single	3	0	
Born in Norway			.532
Yes	70	56	
No	9	5	
Education			.029^*^
No higher education	33	15	
Higher education	46	47	
Employment			.119
Full time	45	46	
Part time	14	5	
At home or on leave	12	8	
Other	7	2	
Gender focus child			.596
Female	34	28	
Male	34	23	

*Notes:* Chi-square results, ^*^ *p* = < .05.

a Fisher's exact test used.

### Materials

The questionnaire comprised of demographic questions, standardized scales on caregivers' psychosocial health and child strengths and difficulties, as well as parenting scales developed to measure parenting behaviours related to the components of ICDP. These were grouped when the scales yielded acceptable psychometric properties. The materials used are listed as follows:

*Activities with the child.* The Parent–Child Activity Scale (Bigner, 1977). This includes 25 items scored on a Likert scale from 1 (never) to 5 (always) (α = .88 at baseline).

*Child's behaviours of watching television and playing computer game:* Caregivers were asked to indicate the number of hours the child spent watching television and playing computer games.

*Positive discipline.* Seven items on the use of positive discipline were created (e.g. “Explaining a better alternative behaviour”). Their format was based on the Conflict Tactic Scale (Straus, 1979), with the caregiver being asked to indicate how frequently they engaged in the behaviours (0, 1–2, 3–10 or more than 10 times). The seven items loaded on one factor in a principal component analysis (PCA) (α = .68). The items were therefore accepted as representing a scale. A summed score that could range from 0 to 105 was created by adding mid-points for the response categories, with a higher score representing more frequent positive discipline.

*Household commotion*. The Confusion, Hubbub, and Order Scale (Matheny, Wachs, Ludwig, & Phillips, 1995). This includes 15 items scored true or false. The summed score can range from 0 to 15 (Cronbach's α = .73). A higher score represents a more chaotic, disorganized and hurried household.

*Happiness with partner.* Drawn from the Dyadic Adjustment Scale (Spanier, 1976). A Visual Analogue Scale (VAS) scored from 0 (extremely unhappy) to 6 (perfectly happy) taken from the Dyadic Adjustment Scale.

*Parenting strategy.* Four items were created to measure caregivers' parenting strategies with a focus on the comprehensive dialogue of the ICDP (e.g. “I adjust myself to my child's interests”), scored from 1 (strongly disagree) to 6 (strongly agree). The items loaded on one factor in a PCA (α = .71 at baseline) and were therefore accepted as representing a scale. A summed score was created that could range from 4 to 24. A higher score represents greater parenting strategies.

*Child management.* Seven items were created to measure caregivers' child management strategies with a focus on the emotional dialogue of the ICDP programme (e.g. “I find it difficult to have emotional conversations with my child”), scored from 1 (agree completely) to 5 (completely disagree). Negatively phrased items were reverse coded, so that a lower score was always better. The items loaded on one factor at baseline in a PCA (Cronbach's α = .64). The items were therefore accepted as representing a scale and an average score for the items was created that could range from 1 to 5.

*Strength and Difficulties Questionnaire* (SDQ; Goodman, 1999). A brief behavioural screening questionnaire about the child. This consists of five subscales (Emotional Symptoms, Conduct Problems, Hyperactivity, Peer Problems, Prosocial) as well as an impact supplement. Three SDQ scores were generated: total difficulties score (the sum of items from the first four subscales, α = .73 at baseline), a prosocial score (α = .75 at baseline) and an impact score.

*Health and quality of life.* SF-36 VAS Scale (Ware, Snow, Kosinski, & Gandek, 1993). Two SF-36 VAS scales were used, scored 0 on the extreme left and 100 on the extreme right.

*Loneliness.* UCLA (University of California, Los Angeles) Loneliness Scale (Russell, 1996). This consists of seven items scored from 1 (hardly ever/ever) to 3 (often). The summed score can range from 7 to 21 (α = .78 at baseline).

*Life satisfaction.* The Satisfaction with Life Scale (Diener, Emmons, Larsen, & Griffin, 1985). This consists of five statements scored from 1 (disagree completely) to 7 (strongly agree). The summed score can range from 5 to 35 (Cronbach's α = .87 at baseline).

*Self-esteem.* The Rosenberg Self Esteem Scale (RSE; Rosenberg, 1965). This consists of 10 items scored from 0 (strongly disagree) to 3 (strongly agree). The summed score can range from 0 to 30 (Cronbach's α = .84 at baseline).

*Self-efficacy.* The Generalized Self-Efficacy Scale (Schwarzer & Jerusalem, 1995). This consists of 10 items scored from 1 (not at all true) to 4 (exactly true). The summed score can range from 10 to 40 (Cronbach's α = .89 at baseline).

*Anxiety and depression*. Hospitalized Anxiety and Depression Scale (HADS; Zigmond & Snaith, 1983). This consists of seven anxiety and seven depression items scored from 0 (not at all) to 3 (very often, most of the time, definitely, very much). Two summed scores were created, one for anxiety (α = .78 at baseline) and one for depression (α = .69 at baseline), each scored from 0 to 21.

### Procedure

The study was approved by the Regional Committee for Medical and Health Research Ethics and the Norwegian Social Science Data Services. The ICDP group completed questionnaires at the first meeting, after the last meeting and 6–12 months later. The comparison group completed the same questionnaires within the same timeline.

### Design and plan of analyses

The study used a 2 (group: ICDP/comparison) × 3 time (pre-ICDP/post-ICDP and 6–12 months follow-up) mixed design. Chi-square and *t*-tests were used to compare the ICDP group and the comparison group on demographic variables and questionnaire scores.

Because of group differences in terms of education, the study used repeated measures analysis of covariance (ANCOVA) with pre-scores as covariate, group (ICDP/comparison) and education (higher education/no higher education) as between-subject factors and time of measurement (post/follow-up) as within-subject factor. The analysis of the questionnaire scores after the ICDP programme and at follow-up hence takes into account the impact of variability in pre-scores between the ICDP and comparison group.

## RESULTS

### Attendance

In the ICDP group, 30 parents (42.9%) attended all meetings, 18 missed one (25.7%), 14 missed two (20%) and eight missed more than two meetings (11.4%). Linear regression analyses showed no significant relationship between the number of sessions attended and change in scores between first and third completion of the questionnaire, except for self-esteem (β = − .263, *p* = .033) and depression (β = .203, *p* = .037). These latter results indicate a greater increase in self-esteem and a greater reduction in depression for the caregivers who missed fewer ICDP sessions.

### Parenting behaviours and child difficulties

Table 2 shows scores for both groups on parental behaviours and child difficulties before and after the ICDP programme, and at follow-up. A significant group effect on parenting strategies indicates a greater increase in scores in the ICDP group from before to after the programme, with the scores of the ICDP group becoming more similar to the comparison group at follow-up (*M*/SD = 18.74/1.97, 19.63/1.69, 18.84/1.98 vs. 19.33/2.30, 18.98/2.59, 18.86/2.52). There was no significant group and time interaction or main effect of time, indicating that changes in scores were maintained at follow-up.

**Table 2:** Scores for the ICDP group and comparison group on parenting behaviours and child difficulties before, after and at follow-up based on repeated measures ANCOVAs with pre-scores as covariate and time of measurement (after/follow-up) as within-subject factor

			Before ICDP		After ICDP		Follow-up										
Measure	Group	N	Mean	SD	Mean	SD	Mean	SD	F Group	*p*		F inter	*p*		F within	*p*	
Parenting strategies	ICDP	62	18.74	1.97	19.63	1.69	18.84	1.98	6.38	.013^*^	.053	1.15	.287	.010	.16	.69	.001
	Comp	57	19.33	2.30	18.98	2.59	18.86	2.52									
Activities (25–125)	ICDP	23	104.74	9.55	105.65	10.62	105.17	9.27	1.98	.166	.039	2.40	.128	.047	1.45	.234	.029
	Comp	31	104.29	8.90	101.23	9.11	103.68	9.02									
Watch TV/play computer games with adult^1^	ICDP	22	2.58	2.66	2.21	1.92	2.09	1.63	5.54	.024^*^	.130	.020	.887	.001	2.15	.151	.055
	Comp	20	1.40	1.18	1.75	1.52	2.01	1.61									
Child management	ICDP	45	2.26	.55	2.07	.50	2.15	.51	12.93	.001^*^	.144	2.56	.113	.032	8.89	.004^*^	.104
	Comp	37	2.11	.48	2.16	.46	2.44	.63									
Positive discipline (0–105)	ICDP	42	44.92	19.12	52.92	24.47	44.93	13.65	1.37	.244	.016	.854	.358	.010	.98	.325	.012
	Comp	47	44.52	21.70	45.61	21.38	43.18	22.76									
SDQ (total difficulties) (0–40)	ICDP	60	8.30	4.09	7.22	4.47	7.67	4.76	3.03	.084	.028	1.22	.272	.011	1.05	.308	.010
	Comp	50	6.10	3.30	6.34	3.88	6.18	3.13									
SDQ prosocial (0–10)	ICDP	61	7.20	2.38	7.47	2.34	7.49	2.20	.014	.906	.000	.001	.975	.000	1.84	.178	.017
	Comp	53	7.83	2.03	7.98	1.81	7.89	1.61									
SDQ impact score (0–10)	ICDP	58	.43	.88	.27	.87	.53	1.59	.125	.724	.001	.728^a^	.395	.007	.59	.442	.005
	Comp	57	.09	.34	.11	.45	.07	.26									

*Notes*: *F* = ANOVA, interaction between group and time, measuring the difference in change scores between the two groups. *p,* probability; 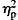, effect size. ^*^ *p* = < .05.

a Please note that the means for “Watching TV and playing computer games with adult” are given in hours.

There was a significant group effect for the amount of time the child spent watching television and playing computer games with an adult. The ICDP group showed a reduction and the comparison group an increase in scores from before to after the programme (*M*/SD = 2.58/2.66, 2.21/1.92, 2.09/1.63 vs. 1.40/1.18, 1.75/1.52, 2.01/1.61) (see Table 2). There was no significant group and time interaction or effect of time, indicating that changes in scores during the programme were maintained at follow-up.

There was a significant group effect on the child management scale (*M*/SD = 2.26/.55, 2.07/.50, 2.15/.51 vs. 2.11/.48, 2.16/.46, 2.44/.63) indicating improved scores for the ICDP group (a lower score represent a greater ability to manage the child). There was also a significant increase in scores from the end of the programme to follow-up, indicating lower proficiency in child management over time, although the scores of the comparison group appear responsible for this overall increase in scores. These main effects were not qualified by a significant group and time interaction (see Table 2).

A group effect on SDQ total difficulties approached significance, indicating a trend towards larger reduction in child difficulties in the ICDP group (*M*/SD = 8.30/4.09, 7.22/4.47, 7.67/4.76 vs. 6.10/3.30, 6.34/3.88, 6.18/3.13) (see Table 2). There was no significant group and time interaction or main effect of time for this measure, indicating that gains achieved during the programme were maintained at follow-up (see Table 2).

### Parental psychosocial measures

Table 3 shows scores for both groups on parental psychosocial measures before and after the ICDP programme, and at follow-up. A significant group effect on loneliness indicates a greater reduction in loneliness in the ICDP group than in the comparison group, with the scores of the ICDP group becoming more similar to the comparison group after the programme and at follow-up (*M*/SD = 12.06/4.44, 11.48/3.76, 11.17/3.60 vs. 11.19/3.39, 11.75/3.86, 11.25/4.19). A group effect for self-efficacy that approached significance indicates a greater improvement in self-efficacy in the ICDP group (*M*/SD = 28.60/5.17, 29.52/5.90, 30.32/4.86 vs. 30.97/5.91, 31.12/5.38, 31.24/7.47). There was no significant group and time interaction or main effect of time on these measures, indicating that changes in scores during the programme were maintained at follow-up (see Table 3).

**Table 3:** Scores for the ICDP group and comparison group on parental psychosocial measures before, after and at follow-up based on repeated measures ANCOVAs with pre-scores as covariate and time of measurement (after/follow-up) as within-subject factor

			Before ICDP		After ICDP		Follow-up										
Measure	Group	N	Mean	SD	Mean	SD	Mean	SD	F Group	*p*		F inter	*p*		F within	*p*	
Commotion (0–15)	ICDP	50	2.64	2.75	2.16	2.34	2.58	2.72	1.49	.225	.015	.920	.340	.009	.11	.745	.001
	Comp	53	1.71	1.98	1.79	1.91	2.57	3.81									
Happiness with partner (0–6)	ICDP	57	3.46	.825	3.65	.79	3.53	.89	1.40	.239	.013	.768	.383	.007	.91	.343	.008
	Comp	56	3.88	1.11	3.79	.97	3.84	1.25									
My health (0–100)	ICDP	67	79.63	14.02	78.70	14.72	78.81	16.26	.034	.853	.000	1.66	.200	.013	6.41	.013^*^	.049
	Comp	62	81.21	14.67	81.94	10.46	80.97	15.83									
My life quality (0–100)	ICDP	67	79.93	11.46	81.34	12.08	78.73	13.88	.001	.972	.000	.028	.867	.000	3.65	.058	.029
	Comp	62	81.61	14.28	82.50	11.83	81.69	15.76									
Loneliness (7–21)	ICDP	63	12.06	4.44	11.48	3.76	11.17	3.60	10.18	.002^*^	.080	.008	.927	.000	2.73	.101	.023
	Comp	59	11.19	3.91	11.75	3.86	11.25	4.19									
Life satisfaction (5–35)	ICDP	66	26.20	5.33	26.59	4.61	26.17	5.08	.953	.331	.008	.022	.882	.000	.21	.645	.002
	Comp	58	27.81	4.94	27.67	5.22	27.48	5.17									
Self-esteem (0–30)	ICDP	51	21.10	3.80	21.29	3.90	21.24	3.73	.234	.630	.002	.039	.843	.000	.82	.368	.008
	Comp	51	21.96	4.86	22.76	4.49	22.25	4.91									
Self-efficacy (10–40)	ICDP	65	28.60	5.17	29.52	5.90	30.32	4.86	3.11	.080	.025	.514	.475	.004	.01	.937	.000
	Comp	59	30.97	5.91	31.12	5.38	31.24	7.47									
Anxiety (0–21)	ICDP	67	5.54	3.64	4.76	3.12	4.64	3.25	1.93	.167	.016	.017	.897	.000	.08	.777	.001
	Comp	59	4.69	3.05	4.34	3.07	4.69	3.81									
Depression (0–21)	ICDP	67	3.10	2.45	3.15	2.67	2.73	2.38	1.20	.276	.010	.219	.641	.002	.78	.379	.006
	Comp	60	3.03	2.56	3.20	2.94	2.88	2.99									

*Notes*: *F* = ANOVA, interaction between group and time, measuring the difference in change scores between the two groups; *p*, probability; 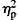, effect size. ^*^ *p* = < .05.

## DISCUSSION

For most measures, the changes from before to 6–12 months after the ICDP programme and the differences between the groups were maintained but at a somewhat lower level. Comparison group scores were often higher than the ICDP group, reflecting largest gains for caregivers with lower initial scores (Sherr et al., 2014). The analyses indicate positive changes in the ICDP group in relation to parenting strategies and child management, and less television viewing and playing computer games in the follow-up period. Extensive television viewing may increase the risks for attention problems (Christakis, Zimmerman, DiGiuseppe, & McCarty, 2004), for leaving school without qualifications (Hancox, Milne, & Poulton, 2005) and for obesity (Øverby, Lillegaard, Johansson, & Andersen, 2004), and time spent playing video games are positively related to increased aggressive behaviour, cognition and affect, as well as decreased empathy and prosocial behaviour (Anderson et al., 2010). A reduction in the time children spend in front of a screen might reflect positive and more active parenting, and the positive effects of the ICDP programme seemed to be sustained over time for parenting strategies.

The decline in self-reported loneliness in the ICDP group during the programme was sustained over time, suggesting that the programme had a continuous beneficial effect on reducing loneliness. Loneliness is often related to psychological difficulties (e.g. Cacioppo, Hughes, Waite, Hawkley, & Thisted, 2006), and Norwegian studies have found that 20% of adults over the age of 30 feel lonely (Thorsen & Clausen, 2009). A reduction in self-reported loneliness is likely to be beneficial for the psychosocial health of parents. The trend in the ICDP group towards a greater increase in self-efficacy indicates that the programme may have had a positive effect on family empowerment, which previously has been associated with decreases in conduct problems in children (Graves & Shelton, 2007). There was a trend towards greater reduction in perceived child difficulties in the ICDP group than in the comparison group which were maintained at follow-up. These effects only approached significance, and it might be that it takes more time before changes in parenting become manifested in the child's behaviour (Vitaro et al., 2001), which may indicate a need for longer-term follow-up studies. Based on the current findings, future studies should examine whether the three-point change cycle suggested by DeGarmo et al. (2004) should be extended with an additional point 2, and thus a cycle with: (1) more effective, including more positive, parenting practices; (2) *decreased loneliness and increased parental self-efficacy*, and finally a reduction in (3) child conduct difficulties and (4) parental depression. The mean scores for child difficulties were higher at follow-up than immediately after the programme, which is in line with the general trend in the data. More intensive training with follow-up sessions may be worth considering in a longer-term implementation plan to sustain early changes and boost initial gains.

The present results should be considered with caution given various limitations. As this was a field study, there was no random allocation to groups. Randomized controlled trials are needed to assess effects of the ICDP programme compared to other programmes and non-receivers by controlling for cofounding factors that might interfere with the results (Duflo, Glennerster, & Kremer, 2008). However, the results from controlled studies where the professionals have been trained for the purpose of the study cannot necessarily be transferred to real field practice. It is therefore necessary to gain knowledge about programme effectiveness in an ordinary field setting (Leichsenring, 2004). Questionnaires completed at home and mailed may not have been completed under standardized conditions, and questionnaires were lengthy possibly resulting in participant fatigue. The level of education differed between the ICDP and comparison group and was controlled for in the analyses. The comparison group had higher completion score than the ICDP group and it might be that parents with higher socioeconomic status are less likely to drop out from the study (Reyno & McGrath, 2006). Reduced participation over the course of the study may have skewed the data towards participants with good follow-up and this will affect the extent to which these findings generalize. The small sample size may have reduced the power to detect significant differences, yet small effect sizes were detected (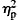 = .045).

Despite the limitations, the findings in this study add evidence to the body of research demonstrating long-term positive effects of parenting programmes on parents, parenting and child behaviour. The significant parent-reported improvements in parenting strategies, some aspects of child management and loneliness immediately after the programme were maintained 6–12 months after the programme. This may point towards initiatives that give priority to preventive family work to benefit children and families. Norway is facing an increase in the number of children and families referred to child protection systems, and concerns over parenting skills is a major prompt (Clausen & Kristofersen, 2008). Research has demonstrated long-term socioeconomic effects of parenting interventions on higher school completion rates, lower welfare dependency, lower crime rates, and gains in productivity (e.g. Reynolds, Temple, Robertson, & Mann, 2001). For example, the High Scope Perry Preschool Project in the USA is estimated to have saved 7.16 dollars for every dollar invested (Temple & Reynolds, 2007). A future evaluation of the ICDP should include observations of parent–child interactions and have a more child-focused design, as the focus of the current study was on parents and based on parental reports. Children should be followed up from childhood to adulthood to see how the effects of publically available parenting programmes such as the ICDP benefit children's long-term development and consequently the society in general, e.g., whether it may reduce the number of children in need of support from health and social services. Initiatives should be taken to increase attendance and include more follow-up sessions, as the results showed greater increase in self-esteem and a greater reduction in depression in ICDP caregivers who missed fewer sessions. Overall, the evaluation demonstrates a sustained long-term benefit on a number of outcomes, thus endorsing the merit of the ICDP with some suggestions for ensuring fidelity to the programme, encouraging full attendance and considering follow-up or re-inoculation to maintain benefits over time.
